# Monomeric C-reactive protein as a biomarker for major depressive disorder

**DOI:** 10.3389/fpsyt.2023.1325220

**Published:** 2024-01-05

**Authors:** Mary G. Hornick, Lawrence A. Potempa

**Affiliations:** College of Science, Health and Pharmacy, Roosevelt University, Schaumburg, IL, United States

**Keywords:** mCRP, C-reactive protein, major depressive disorder, neuroinflammation, biomarker

## Abstract

Neuroinflammation has been postulated to be a key factor in the pathogenesis of major depressive disorder (MDD). With this is mind, there has been a wave of research looking into pro-inflammatory mediators as potential biomarkers for MDD. One such mediator is the acute phase protein, C-reactive protein (CRP). While several studies have investigated the potential of CRP as a biomarker for MDD, the results have been inconsistent. One explanation for the lack of consistent findings may be that the high-sensitivity CRP tests utilized in these studies only measure the pentameric isoform of CRP (pCRP). Recent research, however, has indicated that the monomeric isoform of CRP (mCRP) is responsible for the pro-inflammatory function of CRP, while pCRP is weakly anti-inflammatory. The objective of this minireview is to re-examine the evidence of CRP involvement in MDD with a view of mCRP as a potential biomarker.

## Introduction

Major depressive disorder (MDD) is a significant public health concern, disrupting the lives of millions of individuals worldwide. In the U.S. alone, MDD is the most common mental disorder among adults affecting ~30 million Americans aged 18–65 each year (1)Despite its significance, the pathogenesis of MDD has remained elusive, with multiple mechanisms (e.g., monoamine deficiency, neuroinflammation, hypothalamus-pituitary–adrenal (HPA) axis dysregulation) proposed and investigated. In truth, each theory holds weight as MDD is a multi-factorial disease, and the proposed pathways overlap and feed into each other (2).

One particular mechanism that has gained interest over the last few years is the concept of neuroinflammation leading to MDD. Acute and chronic inflammation, whether from immunotherapy, illness or autoimmunity, is accompanied by the release of pro-inflammatory cytokines which can lead to decreased monoamine synthesis, HPA axis dysregulation and an increase in cytotoxic compounds from activation of the kynurenine metabolism pathway (3–7). Understanding and utilizing the evidence of neuroinflammation in MDD can help researchers and clinicians to identify potential biomarkers early in process and may allow for more individualized treatment.

One such inflammation-related molecule is C-reactive protein (CRP). CRP is an acute phase protein expressed and released mainly by the liver, along with immune cells, endothelial cells and kidneys in response to cytokine signaling at sites of infection or tissue injury (8–11). As an innate immune inflammatory mediator, CRP has been extensively investigated in multiple neuropsychiatric disorders including MDD, anxiety, PTSD and schizophrenia for its potential as a biomarker (12–18). Unfortunately, the high variability of these studies has led to inconclusive or contradictory results (13, 16, 19).

In general, these conflicting conclusions regarding the association between CRP and MDD have been explained as study-to-study variations in accounting for a number of confounding factors such as age, race, obesity, sex, etc. For a comprehensive review of the above factors contributing to high variability of CRP in relation to MDD, see Horn et al. (13). Additionally, it is unclear whether CRP is a cause or a result of MDD. One aspect that has not been investigated, but may help to clarify the relationship, is the concept of utilizing different CRP isoforms. While CRP was initially identified and described as a pentamer with five homologous subunits, it has since been recognized that the protein exists in three distinct isoforms: (1) pentameric CRP (pCRP), (2) monomeric CRP (mCRP) in which a conformational change dissociates the individual subunits from the pentamer, and (3) an intermediary pentameric form that expresses some of the monomeric structural characteristics (pCRP*/mCRP_m_) (20). Each of these isoforms have unique anti-inflammatory or pro-inflammatory functions. To date, there has not been an investigation into the potential of mCRP specifically as a biomarker for MDD. The objective of the present review, therefore, is to apply our new understanding of CRP isoform dynamics and function to re-examine the evidence for the association of CRP with MDD.

## C-reactive protein isoforms

pCRP is an acute phase reactant that is mainly secreted by the liver into the systemic circulation in response to inflammation. As a soluble oligo-protein that increases exponentially in response to acute infection or injury, pCRP has historically been used as a non-specific biomarker of inflammation in acute or chronic diseases (21, 22). This circulating pCRP has been shown to have weak anti-inflammatory functions – decreasing phospholipase A_2_, platelet adhesion factor-induced aggregation of platelets, neutrophil–platelet and-neutrophil adhesion, and decreasing monocyte iNOS production (20, 23). Upon interaction with damaged membranes, however, pCRP binds to lysophophatidylcholine, initiating conversion of pCRP to the intermediary pCRP*/mCRP_m_ isoform and, subsequently, mCRP (24–26). mCRP then acts both at the site of injury/inflammation and distantly via the circulation by association with extracellular lipid rafts (27, 28). Unlike pCRP, mCRP is insoluble in the blood unless protein-or membrane-bound and functions as a pro-inflammatory mediator. mCRP has been shown to increase expression and release of thromboxane A_2_, P-selectin, adhesion molecules, reactive oxygen species, IL-1, IL-6, IL-8 and TNFα (20). In the brain, mCRP has mainly been investigated with respect to infarcts and Alzheimer’s disease (AD). Peripheral mCRP was found to reduce the expression of neuroprotective Apolipoprotein E (ApoE)and decrease the binding of ApoE-LRP1 in the brains of ApoE4 mice (29). Additionally, elevated peripheral mCRP during chronic inflammation leads to decreased CD31 expression and increased phosphorylation of CD31 resulting in cerebrovascular damage. This damage in turn allows for increased T lymphocyte extravasation and the development of pathological markers of AD particularly for carriers of the ApoE4 allele (30, 31). Associations between AD risk and mCRP expression are likewise strongly associated with the existence of previous infarctions. In the areas of previous hemorrhagic or ischemic infarct, high levels of mCRP have been found associated with activated glia and macrophages as well as abnormal neurons expressing p-tau and beta amyloid (32–34). This concentration of mCRP was generally associated with new and/or leaky microvasculature and the surrounding extracellular matrix. Although a majority of the mCRP detected in AD/infarct brains has been at the site of injury, it is important to note that it can be carried via microvasculature to remote sites of the brain. Indeed, in normal areas of the cortex of the AD/infarct brains and controls, mCRP was not found, but it was present in the hippocampal region despite a lack of injury to this area (33). While the previous study only identified small quantities of mCRP in the normal hippocampal neurons, other studies have found weak cytoplasmic expression of mCRP in normal cortical neurons as well (35). In focusing on CRP levels in MDD, Felger et al. demonstrated that plasma CRP correlated with CSF CRP and that high levels of both of these were associated with increased CSF inflammatory markers and the severity of depressive symptoms (36). The above studies indicate that, while pCRP/mCRP is found in higher concentrations at the site of neuronal and neurovascular injury pointing to potential peripheral CRP cross-over of the damaged BBB, there is also some localized expression within the normal brain and CSF circulation.

## Role of mCRP in MDD

As mentioned previously, the current data regarding the association of CRP levels with MDD is conflicting. While a number of studies have found a significant correlation between peripheral CRP levels and the development of or existent MDD, several have found no such association, particularly when adjusting for confounding factors. In a meta-analysis conducted by Horn et al., only 23% of studies out of 35 analyses reported a statistically significant relationship between depression and CRP (13). Similarly, Orsolini et al. analyzed 56 studies, with 26 (46%) showing no association between CRP and depression (16). It should be noted that, in both of these reviews, a large number of studies initially did demonstrate a significant association, but that significance disappeared when adjusted for age, race, sex, weight/BMI, depression subtype, cardiovascular risk factors and/or chronic medical conditions. Adding to the confusion is the degree and duration of depression that was measured, although, in general, it was found that higher CRP was associated with greater severity of symptoms, rather than an increased risk of developing MDD.

Another major point of variability between previous reports is the definition of what exactly is meant by ‘high CRP levels’. For those studies that indicated how CRP was measured, the vast majority utilized high sensitivity CRP (hsCRP) assays. The purported advantage of hsCRP assays is that they are able to detect low CRP levels in the blood (~0.007 mg/L vs. 3–5 mg/L for conventional CRP assays). As such, these hsCRP assays have been promoted and widely utilized as indications of disease risk, particularly for cardiovascular conditions (37, 38). While the U.S. Food and Drug Administration recognizes serum levels of CRP >10 mg/L as diagnostic of non-specific inflammation, there is no consensus on serum levels between 1 and 10 mg/L. Indeed, many studies dismiss the CRP measurements that exceed their arbitrary range, i.e., ‘undetectable’ and >10 mg/L (13). The studies of CRP in depression, therefore, do not all utilize the same definitions of ‘high’, ‘low’, or ‘mid’ CRP levels. Indeed, ranges of CRP ‘positivity’ that were included in various studies include as low as 0.005 mg/L and as high 12 mg/L, with some indicating >3 mg/L as ‘high’ and others setting the cutoff at >1 mg/L (39–42). This practice adds to the confusion and lack of replicability of these studies, which may at least partially explain the high variation both intra-and inter-group. Finally, and most relevant to the present review, hsCRP does not distinguish between CRP isoforms (43).

As noted above, pCRP is present at much higher levels in the blood and exhibits weak anti-inflammatory properties despite being an overall indicator of inflammation, while mCRP is present at lower levels and exhibits pro-inflammatory properties. While there have been a couple of studies that examined the correlation between mCRP and pCRP concentrations in plasma or serum, the results have been inconclusive. In one study measuring CRP in patients with COVID-19, a weak positive correlation (correlation coefficient r = 0.377) was found between elevated mCRP and pCRP levels (44). Furthermore, it was found that log_mCRP/pCRP only correlated with two of the established severity markers for COVID-19, while mCRP levels significantly correlated with five markers for severity. In a study examining the potential of mCRP as a biomarker for Adult Onset Still’s Disease (AOSD), however, no correlation was found between mCRP and pCRP (R^2^ = 0.08) although high levels of mCRP and the mCRP (x1000)/pCRP ratio were significantly higher in AOSD patients than those with rheumatoid arthritis, polymyalgia rheumatica, or infection (45). In revisiting previous research, therefore, one would anticipate that a sample which does not distinguish between CRP isoforms would naturally contain more pCRP, potentially masking any impact of mCRP on disease state or disease state on mCRP. While it is now possible to measure peripheral and central mCRP via ELISA, this direct method has yet to be utilized in MDD patients (32–35, 46). Given the current lack of studies directly measuring mCRP in MDD, we can only estimate high levels of mCRP in previous MDD studies indirectly by looking at downstream inflammatory markers that are typically enhanced due to mCRP.

**Figure 1:**
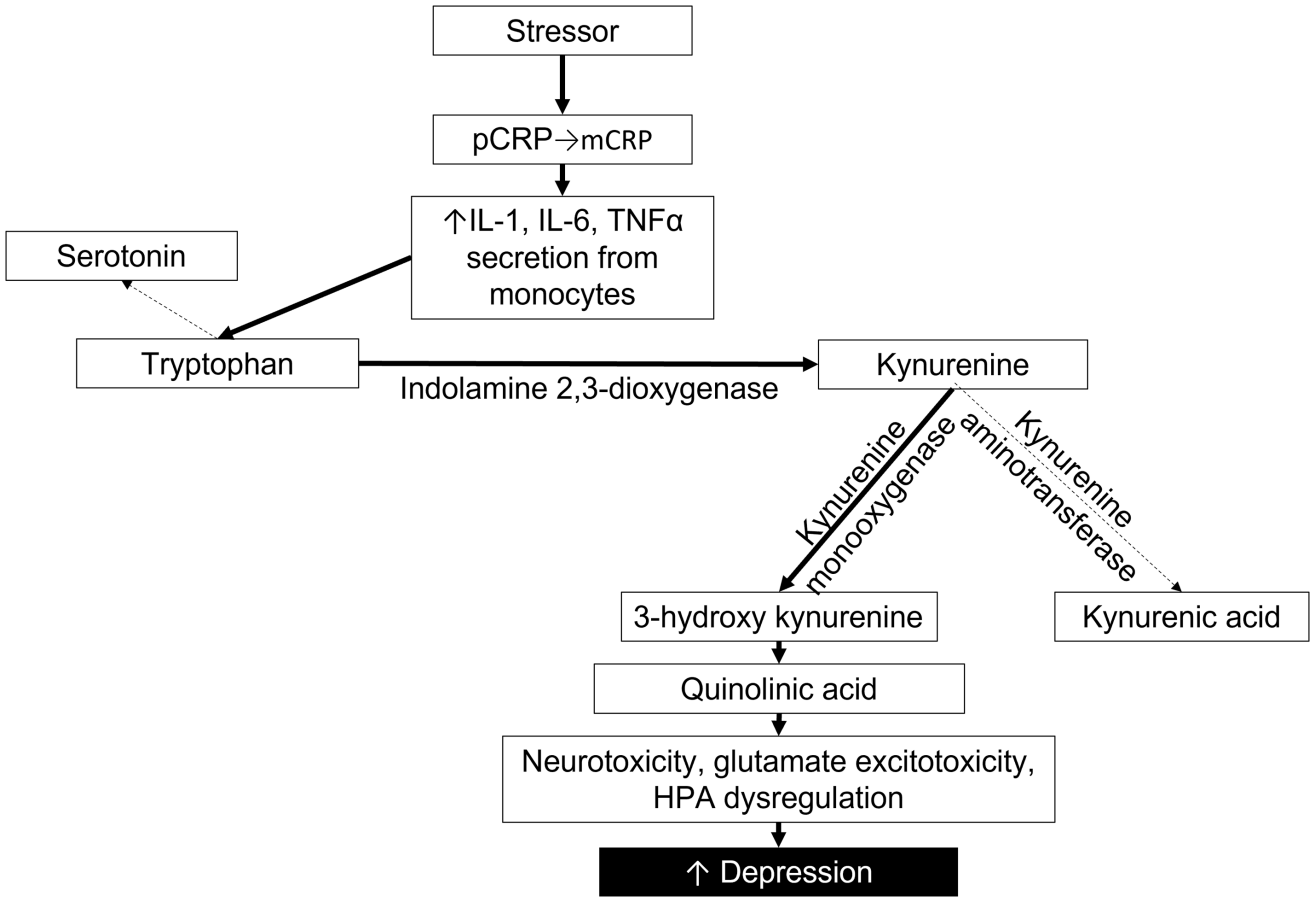
Proposed pathway depicting the impact of mCRP on cytokines, tryptophan metabolism, the kynurenine pathway and depression severity. Solid lines indicate pathway enhanced by mCRP.

mCRP increases the expression and release of adhesion molecules and pro-inflammatory cytokines IL-1, IL-6, IL-8, and TNFα (20). In a meta-analysis by Howren et al., positive associations were found between depression and IL-1, IL-6 and CRP (47). As these cytokines are associated with mCRP, but not pCRP, it is likely that the positive association between MDD and CRP levels reflects a higher concentration of mCRP. In addition to the cytokines directly released by mCRP, we can also look to studies that investigate the downstream metabolic pathways that are stimulated by high levels of CRP and pro-inflammatory cytokines. In particular, there is a strong association between high CRP, TNFα, and IL-10 with the kynurenine metabolic pathway (7). Both psychological stress and physical inflammation result in an increased breakdown of tryptophan (TRP), shunting TRP away from serotonin synthesis and toward the kynurenine (KYN) pathway (48). Interferon gamma (INFγ), IL-6 and TNFα activate indolamine 2,3-dioxygenase (IDO) to metabolize TRP into KYN. KYN is then further metabolized either by kynurenine amino transferase into kynurenic acid or by kynurenine monooxygenase into 3-hydroxy kynurenine (3HK). 3HK is then degraded into quinolinic acid, an NMDA agonist associated with neurotoxicity, glutamate excitotoxicity, dysregulation of the HPA axis, and, ultimately, depression (49). Several studies have utilized the KYN/TRP ratio as an indirect measure of inflammation and IDO activity. KYN/TRP is strongly associated with elevated hsCRP levels and the development or presence of MDD (3, 7, 50–53). Thus, by re-evaluating studies in which measurements of pro-inflammatory cytokines and KYN/TRP were established along with hsCRP, we are able to indirectly estimate the activity of mCRP (Figure 1).

## Discussion

While neuroinflammation plays a key role in the development of certain types of MDD, it is important to acknowledge that not all MDD presents in the same way or necessarily has the same pathogenesis. This is evidenced by not only the symptoms but also by the response of patients to various treatments. While a large number of depressed patients respond to traditional antidepressant medication like selective serotonin reuptake inhibitors (SSRIs) or monoamine oxidase inhibitors (MAOIs), some do not. The response to SSRIs or non-SSRIs is at least partially dependent on gene-drug interactions, such as with CYP450 liver enzymes and their genetic variants. By utilizing inflammatory biomarkers, such as cytokines, KYN/TRP, and mCRP, it may be possible to determine which patients would instead benefit from anti-inflammatory medication or NMDA antagonists.

Indeed, associations have already been noted between different types of MDD and relative levels of inflammatory markers. Hickman et al. found that patients with ‘atypical’ MDD, characterized by vegetative symptoms, hypersomnia and hyperphagia, were approximately 2 times as likely to have higher hsCRP levels than healthy controls or those with ‘typical’ MDD, characterized by depressed mood, sleep disturbance, and decreased appetite (42). Higher hsCRP levels have also been associated with increased severity of depressive symptoms (16, 40, 54). It has been postulated that the approximately 30% of patients who do not respond to traditional treatment may coincide with the ~30% of patients who present with low grade inflammation as determined by CRP levels (16). By screening patients for neuroinflammation using peripheral biomarkers, we may be able to identify certain patients who may benefit from the inclusion of an anti-inflammatory agent along with an antidepressant. In fact, this concept of identifying peripheral biomarkers of central inflammation is certainly not new, although the ability to do so has recently improved due to the development of protocols for measuring neuron-and astrocyte-derived extracellular vesicles (55). Extracellular vesicles (EVs) are nanoscale, lipid-enclosed vesicles that are naturally secreted from a number of cells. While they do not contain functional nuclei, they may contain proteins, lipids, nucleic acids or other biomolecules and can have an effect on other cells. Neuron-derived EVs that contain miR-9-5p, for example, are capable of inducing M2 polarization of microglia and the subsequent release of inflammatory cytokines IL-1β, IL-6 and TNF-α. Due to their nanoscale lipid structure, these brain-derived EVs can easily transverse the blood–brain barrier and end up in the peripheral circulation where they may be measured as an indication of neuroinflammation (55). The addition of mCRP measurements to existing protocols may help to identify the severity of the inflammation.

Future studies are needed to determine whether mCRP levels correlate better with MDD than hsCRP levels. If indeed they do, mCRP may prove to be a novel biomarker for treatment-resistant or immune-associated MDD.

## Author contributions

MH: Conceptualization, Data curation, Writing – original draft, Writing – review & editing. LP: Conceptualization, Supervision, Writing – review & editing.
